# Exploration of nonclassical symmetries and exact solutions to the (4+1)-dimensional Boiti–Leon–Manna–Pempinelli equation

**DOI:** 10.1038/s41598-025-20839-4

**Published:** 2025-10-05

**Authors:** Mati ur Rahman, Sonia Akram, Muhammad Asif

**Affiliations:** 1 Department of Mathematics and Statistics, College of Sciences, Imam Mohammad Ibn Saud Islamic University (IMSIU), Riyadh, Saudi Arabia; 2 Department of Mathematics, Faculty of Science, University of Gujrat, Gujrat, 50700 Pakistan

**Keywords:** (4 + 1)-Dimensional Boiti–Leon–Manna–Pempinelli, Nonclassical symmetry analysis, Nonlinear partial differential equations, Exact solutions, Graphical analysis, Materials science, Mathematics and computing

## Abstract

This paper presents a complete nonclassical symmetry analysis of the nonlinear integrable model known as the (4 + 1)-dimensional Boiti–Leon–Manna–Pempinelli (4D-BLMP) equation. The analysis is divided into two parts. The first part involves constructing systems of nonlinear partial differential equations for the determining equations based on the dimensions of the model. Five distinct cases of these systems are examined and solutions to these systems are found, leading to the creation of various new nonclassical symmetries. The second part focuses on classifying the developed unknown functions using the constructed nonclassical symmetries and their invariant formulations. These classified functions are then applied to obtain a range of new explicit exact solutions to the model. The paper also includes a graphical analysis of the dynamical behavior of these solutions, taking into account special parameter values. The results highlight the existence of various wave structures in the 4D-BLMP equation, setting it apart from other models that lack non-singular complexiton solutions. The analysis of higher-dimensional nonlinear integrable equations is essential because such models capture complex wave phenomena arising in mathematical physics, fluid dynamics, and optical systems. In particular, understanding their exact and nonclassical solutions provides deeper insight into the underlying dynamics and supports the development of effective analytical and numerical techniques.

## Introduction

The intrinsic nonlinearity of environmental physical problems has sparked a great deal of interest in nonlinear dynamical models among researchers. These models use differential equations to demonstrate a wide domain of physical phenomena and have implications in different technological and scientific fields, involving optics^1^, ocean engineering^2^, quantum mechanics^3^, fluid dynamics^4^, mechanical engineering^5^, cosmology^6^, and others. Differential equations, a branch of mathematics, are utilized to represent the evolution of physical systems over space or time.

Exact solutions are extremely significant across physics, mathematics, and engineering because they offer comprehensive and precise representations of systems without relying on approximations^7^. These solutions provide profound understanding of the underlying behavior of models and phenomena and are frequently presented in closed, analytical forms. For instance, the precise solution to the equations of motion under gravity in physics explains planetary orbits and identifies the fundamental principles that underlie them^8^. This clarity makes it possible to comprehend theory more thoroughly, which can result in more extensive generalizations and discoveries. When exact forms are too difficult or impossible to get, numerical and approximation approaches are frequently used, and exact answers are also essential standards for evaluating their accuracy. Approximations are compared to known exact results to help researchers assess how reliable their models are. A level of generality that numerical solutions could obfuscate is also provided by precise solutions, which offer symbolic expressions that illustrate the interactions between various variables and factors. They are also crucial instruments for theoretical development, aiding in the creation of new frameworks and the testing of hypotheses. Because they don’t require recalculation, exact solutions are frequently computationally efficient, saving time and money^9–11^.

Integrable models play an essential role in mathematical physics, specifically in the study of exactly solvable problems and nonlinear systems. These models are unique because they offer exact analytical solutions, frequently in the form of closed expressions. In general, a system is said to be integrable if it has as many conserved quantities (integrals of motion) as degrees of freedom, which usually permits the full solution^12^. This property makes integrable models effective tools for comprehending intricate dynamics in a mathematically controlled and rigorous way. One of the most impressive characteristics of integrable systems is their capacity to elaborate nonlinear phenomena without resorting to approximations. For example, solitons, localized, stable waves that retain their shape during propagation and after interactions, emerge naturally from integrable equations such as the nonlinear Schrödinger equation and the Korteweg-de Vries (KdV) equation^13^. These solutions have significant applications ranging from quantum field theory to fluid dynamics and optics. Furthermore, integrable models are frequently idealized frameworks that allow for the study of more complex, non-integrable systems.

The (4+1)-dimensional Boiti–Leon–Manna–Pempinelli (BLMP) equation is an essential extension of traditional integrable systems into higher dimensions^14^. Originally presented in lower dimensions, the BLMP equation is part of a class of nonlinear partial differential equations (NLPDEs) that explain intricate wave processes and possess rich mathematical characteristics, including soliton solutions and integrability. Its extension to five dimensions four spatial and one temporal improves its ability to system more realistic physical models, especially in the fields like nonlinear optics, plasma physics, and fluid dynamics, where multidimensional effects cannot be ignored. The investigation of integrable systems has long been central to comprehending nonlinear wave process and their characteristics in distinct scientific fields, including plasma physics, nonlinear optics, and fluid dynamics. In this paper, the 4D-BLMP equation, describing complex interactions in a system with four spatial and one temporal dimension, is considered in the following form^14,15^:

1 $$\begin{aligned} \frac{\partial ^2 \Theta }{\partial t \partial y}+\frac{\partial ^2 \Theta }{\partial t \partial z}+\frac{\partial ^2 \Theta }{\partial t \partial s}+\alpha \bigg [\frac{\partial ^4 \Theta }{\partial y \partial x^3}+\frac{\partial ^4 \Theta }{\partial z \partial x^3}+\frac{\partial ^4 \Theta }{\partial s \partial x^3}\bigg ]+\beta \bigg [\frac{\partial \Theta }{\partial x}(\frac{\partial ^2 \Theta }{\partial y \partial x}+\frac{\partial ^2 \Theta }{\partial z \partial x}+\frac{\partial ^2 \Theta }{\partial s \partial x})+\frac{\partial ^2 \Theta }{\partial x^2}(\frac{\partial \Theta }{\partial y}+\frac{\partial \Theta }{\partial z}+\frac{\partial \Theta }{\partial s})\bigg ]=0, \end{aligned}$$

The importance of the 4D-BLMP equation lies mainly in its integrable feature, which means it satisfies a large variety of exact solutions. These exact solutions are more than simply mathematical oddities; they reflect stable, localized structures that mimic the actual behavior of nonlinear media in the real world and can spread over time without altering shape. Understanding the movement and interaction of energy, information, or disturbances in complex settings is aided by the study of such solutions. Xu and Wazwaz^15^ introduced the aforementioned nonlinear model, which has been examined using a variety of methodologies. Xu and Wazwaz used the Bell polynomial approach to obtain the aforementioned novel model’s bilinear representation, bilinear Bäcklund transformation, Lax pair, and infinite conservation laws. They also showed that the model had the Painlevé property.

It is worth highlighting that when $\Theta =\Theta (x, y, t)$, $\alpha =1,~\beta =-3$, then Eq. (1) reduces to the following $(2+1)$-dimensional BLMP equation^16,17^:

2 $$\begin{aligned} \frac{\partial ^2 \Theta }{\partial t \partial y}+\frac{\partial ^4 \Theta }{\partial y \partial x^3}-3\frac{\partial \Theta }{\partial x}\frac{\partial ^2 \Theta }{\partial y \partial x}-3\frac{\partial ^2 \Theta }{\partial x^2}\frac{\partial \Theta }{\partial y}=0. \end{aligned}$$

Additionally, for $\Theta =\Theta (x,y,z,t)$, $\alpha =1$, and $\beta =-3$, Eq. (1) reduces to the following $(3+1)$-dimensional BLMP equation^18,19^:

3 $$\begin{aligned} \frac{\partial ^2 \Theta }{\partial t \partial y}+\frac{\partial ^2 \Theta }{\partial t \partial z}+\frac{\partial ^4 \Theta }{\partial y \partial x^3}+\frac{\partial ^4 \Theta }{\partial z \partial x^3}-3\bigg [\frac{\partial \Theta }{\partial x}(\frac{\partial ^2 \Theta }{\partial y \partial x}+\frac{\partial ^2 \Theta }{\partial z \partial x})+\frac{\partial ^2 \Theta }{\partial x^2}(\frac{\partial \Theta }{\partial y}+\frac{\partial \Theta }{\partial z})\bigg ]=0. \end{aligned}$$

The ability to obtain exact solutions for such systems is significant for both practical applications and theoretical understanding. Specifically, the higher-dimensional nature of the equation presents novel effects that do not appear in lower-dimensional models, including higher-order singularities of intricate soliton interactions and a greater variety of wave phenomena’s. In lower-dimensional environments, these structures can result in more detailed representations of physical systems that were previously unclear. The integrability of these equations enables researchers to find explicit solutions, offering insights into the nonlinear behavior of waves that would otherwise be challenging to analyze. The 4D-BLMP equation is related to a larger class of nonlinear PDEs that describe the dynamics of wave-like phenomena. There are different techniques to addressing nonlinear differential models, which involves inverse scattering method^20^, Bäcklund transformation^21^, the Darboux transform^22^ and Hirota bilinear form^23^, applying the linear superposition principle^24^ and utilizing symbolic computations to obtain rational wave solutions. The study of NLPDEs often involves finding solutions that are invariant under certain transformations. One of the most significant approaches for solving PDEs using symmetry methods is Lie’s classical symmetry analysis, which involves finding continuous transformations that leave the equation invariant^25^. However, while the classical Lie symmetry method has proven effective for many types of PDEs, it may not always be sufficient for solving more complex or nonlinear equations, particularly in higher dimensions. To overcome these limitations, several generalizations of Lie’s classical method have been introduced, including the generalized conditional symmetries method^26^, the method of heir equations^27^, the direct method^28^, the nonclassical symmetry reduction method^29^, and the B-determining equations method^30^. Among these methods, the nonclassical symmetries method has gained significant popularity and is widely regarded as one of the most effective approaches for solving nonlinear PDEs.

The concept of nonclassical symmetry, often referred to as conditional symmetry, was originally formulated by Bluman and Cole^31^. Unlike the standard Lie symmetry approach, this technique allows for a wider range of admissible transformations by enforcing invariance not only of the governing equation itself but also of the corresponding invariant surface condition together with its differential consequences. In contrast to the classical method, which focuses exclusively on the invariance of the PDE itself, the nonclassical framework requires simultaneous invariance of both the equation and the constraint manifold defined by the invariant surface condition. This additional requirement often leads to reductions and solutions that cannot be obtained by standard Lie techniques. Over the past decades, the nonclassical method has proven invaluable in the analysis of integrable and nonlinear models, particularly in higher-dimensional settings where the complexity of the system limits the effectiveness of classical symmetry methods^32^. In the context of the 4D-BLMP equation, the nonclassical approach provides a systematic procedure for generating determining equations whose solutions yield new symmetry transformations. These, in turn, allow the derivation of a wide range of exact solutions, thereby deepening our understanding of the nonlinear dynamics and rich wave structures associated with the model. Moreover, the practical implementation of the nonclassical method often relies on symbolic computational tools such as *Maple* and specialized packages like SADE, which significantly streamline the derivation and solution of the determining equations. By combining rigorous theoretical analysis with advanced computational techniques, the nonclassical symmetry method offers a powerful framework for uncovering novel solution families such as solitary waves, complexitons, and multi-dimensional structures that are otherwise inaccessible through classical approaches.

The remaining content of our manuscript is structured as follows: “Generalized symmetries” describes the comprehensive analysis of nonclassic symmetry to the proposed 4D-BLMP equation. “Invariant structures via conditional symmetries, determination of auxiliary functions, analytical solutions, and illustrative plots” provides the symmetry reductions, invariant solutions, as well as graphical descritions of the gained solutions. Finally, we illustrate our concluding remark in “Conclusion”.

## Generalized symmetries

In this section, we classify the generalized symmetries of the governing equation. The following gives the operator for non-classical symmetry:

4 $$\begin{aligned} \Psi =\Upsilon ^{1}\frac{\partial }{\partial t} +\Upsilon ^{2}\frac{\partial }{\partial x}+ \Upsilon ^{3} \frac{\partial }{\partial y}+\Upsilon ^{4} \frac{\partial }{\partial z}+\Upsilon ^{5}\frac{\partial }{\partial s}+ \delta \frac{\partial }{\partial \Theta }. \end{aligned}$$

In this context, $\Upsilon ^{m}$ and $\delta$ depend on the variables *t*, *x*, *y*, *z*, *s*,  and $\Theta$ for $m=1,\ldots ,5$. Additionally, the surface condition that remains unchanged is given by,

5 $$\begin{aligned} & \Gamma =\Upsilon ^{1}\frac{\partial \Theta }{\partial t}+ \Upsilon ^{2}\frac{\partial \Theta }{\partial x}+\Upsilon ^{3}\frac{\partial \Theta }{\partial y}+\Upsilon ^{4}\frac{\partial \Theta }{\partial z}+\Upsilon ^{5}\frac{\partial \Theta }{\partial s}-\delta =0. \end{aligned}$$

The equation that defines the generalized symmetries is,

6 $$\begin{aligned} & \Upsilon ^{[2]} \Gamma \mid _{Eq. (1)=0,\; \Gamma =0}=0, \Upsilon ^{[1]} \Gamma \mid _{Eq. (1)=0,\; \Gamma =0}=0, \end{aligned}$$

where the regular first and second prolongations of operator $\Psi$ are $\Psi ^{[1]}$ and $\Psi ^{[2]}$ respectively. There are five possible cases and the analysis of generalized symmetries for every case is provided here.

Case 1: Let us analyze the first condition. In this situation, we assume $\Upsilon ^{1}\ne 0$, specifically taking $\Upsilon ^{1}=1$. After performing the necessary simplifications, the corresponding equations obtained are given below:

7 $$\begin{aligned} {\left\{ \begin{array}{ll} \Upsilon ^{2}_{\Theta }= \Upsilon ^{3}_{\Theta }= \Upsilon ^{4}_{\Theta }=\Upsilon ^{5}_{\Theta }= \delta _{\Theta \Theta }=0, \\ \Upsilon ^{2}_{x}= \Upsilon ^{4}_{x}=\Upsilon ^{5}_{x}=0, \\ \Upsilon ^{4}_{t}=\Upsilon ^{5}_{t}=0, \\ \Upsilon ^{2}_{t}-3\Upsilon ^{3}_{x}=0, \\ \Upsilon ^{3}(\Upsilon ^{2}_{s}+\Upsilon ^{2}_{y}+\Upsilon ^{2}_{z})-\Upsilon ^{2}(\Upsilon ^{3}_{s}+\Upsilon ^{3}_{y}+\Upsilon ^{3}_{z})=0, \\ (\Upsilon ^{5}-1)(\Upsilon ^{2}_{s}+\Upsilon ^{2}_{y}+\Upsilon ^{2}_{z})-\Upsilon ^{2}(\Upsilon ^{5}_{s}+\Upsilon ^{5}_{y}+\Upsilon ^{5}_{z})=0, \\ (\Upsilon ^{4}-1)(\Upsilon ^{2}_{s}+\Upsilon ^{2}_{y}+\Upsilon ^{2}_{z})-\Upsilon ^{2}(\Upsilon ^{4}_{s}+\Upsilon ^{4}_{y}+\Upsilon ^{4}_{z})=0, \\ \Upsilon ^{3}_{xx}-\delta _{xu}=0, \\ (\Upsilon ^{5}-1)(\Upsilon ^{2}_{s}+\Upsilon ^{2}_{y}+\Upsilon ^{2}_{z})-\Upsilon ^{2}(\Upsilon ^{5}_{s}+\Upsilon ^{5}_{y}+\Upsilon ^{5}_{z})+\Upsilon ^{2}(1-\Upsilon ^{5})(3\Upsilon ^{3}_{x}-\Upsilon ^{2}_{t})=0, \\ \Upsilon ^{2}(\delta _{su}+\delta _{yu}+\delta _{zu})-3\Upsilon ^{2}(\Upsilon ^{3}_{xs}+\Upsilon ^{3}_{xy}+\Upsilon ^{3}_{sz})+(\Upsilon ^{2}_{s}+\Upsilon ^{2}_{y}+\Upsilon ^{2}_{z}) (3\Upsilon ^{3}_{x}-\delta _{u})=0, \\ \Upsilon ^{3}_{t}-\delta _{x}=0, \\ \Upsilon ^{3}_{x}+\delta _{u}=0, \\ \Upsilon ^{2}(\delta _{st}+\delta _{yt}+\delta _{zt})-\delta _{t}(\Upsilon ^{2}_{s}+\Upsilon ^{2}_{y}+\Upsilon ^{2}_{z})+\Upsilon ^{2}\delta _{t}(3\Upsilon ^{3}_{x}-\Upsilon ^{2}_{t})+\Upsilon ^{2}\delta ^{2}_{x}=0, \\ \Upsilon ^{2}(1-\Upsilon ^{4})(\beta \delta _{xx}+\delta _{tu})=0, \\ \Upsilon ^{2}(1-\Upsilon ^{5})(\beta \delta _{xx}+\delta _{tu})=0, \\ \Upsilon ^{2}(\delta _{s}+\delta _{y}+\delta _{z})-\delta (\Upsilon ^{2}_{s}+\Upsilon ^{2}_{y}+\Upsilon ^{2}_{z})=0, \\ \Upsilon ^{2}[(\delta _{su}+\delta _{yu}+\delta _{zu})-(\Upsilon ^{2}_{st}+\Upsilon ^{2}_{ty}+\Upsilon ^{2}_{zt})]+(\Upsilon ^{2}_{t}-\delta _{u})(\Upsilon ^{2}_{s}+\Upsilon ^{2}_{y}+\Upsilon ^{2}_{z})-(\Upsilon ^{2})^{2}\delta _{tu}=0, \\ \Upsilon ^{2}[(\delta _{sx}+\delta _{yx}+\delta _{zx})-(\Upsilon ^{3}_{st}+\Upsilon ^{3}_{yt}+\Upsilon ^{3}_{zt})]+\Upsilon ^{2}\delta _{u}(2\delta _{x}-\Upsilon ^{3}_{t})+\Upsilon ^{3}_{t}\Upsilon ^{2}(\Upsilon ^{2}_{t}-2\Upsilon ^{3}_{x})=0, \\ \Upsilon ^{2}[(\delta _{su}+\delta _{yu}+\delta _{zu})-(\Upsilon ^{3}_{sx}+\Upsilon ^{3}_{yx}+\Upsilon ^{3}_{zx})]+(\Upsilon ^{2}_{s}+\Upsilon ^{2}_{y}+\Upsilon ^{2}_{z})(\Upsilon ^{3}_{x}-\delta _{u})-\Upsilon ^{2}[(\Upsilon ^{3}_{x})^{2}-(\delta _{u})^{2}]=0. \end{array}\right. } \end{aligned}$$

After solving the governing equations, the following expressions are obtained: $\Upsilon ^{1}=1,\; \Upsilon ^{2}={\mathcal {A}}(s, y, z),\; \Upsilon ^{3}={\mathcal {B}}(s, y, z),\; \Upsilon ^{4}={\mathcal {C}}(s, y, z),\; \Upsilon ^{5}={\mathcal {D}}(s, y, z), \; \delta ={\mathcal {E}}(s, y, z, t).$ Accordingly, the symmetry operator associated with this case takes the form:

8 $$\begin{aligned} \Psi =\Upsilon ^{1}\partial t +\Upsilon ^{2}\partial x+ \Upsilon ^{3} \partial y+\Upsilon ^{4}\partial z+\Upsilon ^{5}\partial s+ \delta \partial \Theta . \end{aligned}$$

This corresponds to a linear combination (LC) of the nonclassical symmetries. By plugging the obtained values of $\Upsilon ^{1},\ \Upsilon ^{2},\ \Upsilon ^{3},\ \Upsilon ^{4},\ \Upsilon ^{5}$ and $\delta$, the resulting expression is derived as follows:

9 $$\begin{aligned} \Psi =\partial t +{\mathcal {A}}(s, y, z)\partial x+ {\mathcal {B}}(s, y, z) \partial y+{\mathcal {C}}(s, y, z)\partial z +{\mathcal {D}}(s, y, z)\partial s+ {\mathcal {E}}(s, y, z, t) \partial \Theta , \end{aligned}$$

where ${\mathcal {A}}(s, y, z),\; {\mathcal {B}}(s, y, z),\; {\mathcal {C}}(s, y, z),\; {\mathcal {D}}(s, y, z)$ and ${\mathcal {E}}(s, y, z, t)$ are specified functions.

Case 2: Let us now examine the second condition. In this case, we set $\Upsilon ^{1}=0$ and $\Upsilon ^{2}\ne 0$, specifically choosing $\Upsilon ^{2}=1$. After carrying out the necessary simplifications, the governing equations reduce to the following form:

10 $$\begin{aligned} {\left\{ \begin{array}{ll} \Upsilon ^{3}_{\Theta }= \Upsilon ^{4}_{\Theta }=\Upsilon ^{5}_{\Theta }= \delta _{ \Theta }=0, \\ \Upsilon ^{3}_{x}= \Upsilon ^{4}_{x}=\Upsilon ^{5}_{x}= \delta _{x}=0, \\ \Upsilon ^{3}_{t}= \Upsilon ^{4}_{t}=\Upsilon ^{5}_{t}=0, \\ \Upsilon ^{3}_{s}+\Upsilon ^{3}_{y}+\Upsilon ^{3}_{z}=0, \\ \Upsilon ^{4}_{s}+\Upsilon ^{4}_{y}+\Upsilon ^{4}_{z}=0, \\ \Upsilon ^{5}_{s}+\Upsilon ^{5}_{y}+\Upsilon ^{5}_{z}=0, \\ \delta _{ts}+\delta _{ty}+\delta _{tz}=0, \\ \delta _{s}+\delta _{y}+\delta _{z}=0. \end{array}\right. } \end{aligned}$$

By solving these governing equations, we obtain the following results: $\Upsilon ^{2}=1,\; \Upsilon ^{3}={\mathcal {A}}(s, y, z),\; \Upsilon ^{4}={\mathcal {B}}(s, y, z),\; \Upsilon ^{5}={\mathcal {C}}(s, y, z),\; \delta ={\mathcal {D}}(s, y, z, t).$ The corresponding symmetry operator in this case is given by:

11 $$\begin{aligned} \Psi =\Upsilon ^{2}\partial x+ \Upsilon ^{3} \partial y+\Upsilon ^{4}\partial z+\Upsilon ^{5}\partial s+ \delta \partial \Theta . \end{aligned}$$

This corresponds to a linear combination of nonclassical symmetries. Substituting the values of $\Upsilon ^{2},\; \Upsilon ^{3},\; \Upsilon ^{4},\; \Upsilon ^{5}$ and $\delta$ yields the following result:

12 $$\begin{aligned} \Psi =\partial x+ {\mathcal {A}}(s, y, z) \partial y+{\mathcal {B}}(s, y, z)\partial z +{\mathcal {C}}(s, y, z)\partial s+ {\mathcal {D}}(s, y, z, t) \partial \Theta , \end{aligned}$$

where ${\mathcal {A}}(s, y, z),\; {\mathcal {B}}(s, y, z),\; {\mathcal {C}}(s, y, z)$ and ${\mathcal {D}}(s, y, z, t)$ are specified functions.

Case 3: Let us now consider the third condition. Here, we take $\Upsilon ^{1}=0,\; \Upsilon ^{2}=0$ and assume $\Upsilon ^{4}\ne 0$, in particular $\Upsilon ^{4}=1$. After applying the necessary simplifications, the reduced equations are obtained in the following form:

13 $$\begin{aligned} {\left\{ \begin{array}{ll} \Upsilon ^{3}_{\Theta }=\Upsilon ^{5}_{\Theta }= \delta _{ \Theta }=0, \\ \Upsilon ^{3}_{x}=\Upsilon ^{5}_{x}= \delta _{x}=0, \\ \Upsilon ^{3}_{t}=\Upsilon ^{5}_{t}=0, \\ \Upsilon ^{3}_{s}+\Upsilon ^{3}_{y}+\Upsilon ^{3}_{z}=0, \\ \Upsilon ^{5}_{s}+\Upsilon ^{5}_{y}+\Upsilon ^{5}_{z}=0, \\ \delta _{ts}+\delta _{ty}+\delta _{tz}=0, \\ \delta _{s}+\delta _{y}+\delta _{z}=0. \end{array}\right. } \end{aligned}$$

By solving these governing equations, the following results are obtained: $\Upsilon ^{3}={\mathcal {A}}(s, y, z),\; \Upsilon ^{4}=1,\; \Upsilon ^{5}={\mathcal {B}}(s, y, z),\; \delta ={\mathcal {C}}(s, y, z, t).$ The symmetry operator corresponding to this case can be expressed as:

14 $$\begin{aligned} \Psi = \Upsilon ^{3} \partial y+\Upsilon ^{4}\partial z+\Upsilon ^{5}\partial s+ \delta \partial \Theta . \end{aligned}$$

This corresponds to a linear combination of the nonclassical symmetries. Substituting the values of $\Upsilon ^{3},\; \Upsilon ^{4},\; \Upsilon ^{5}$ and $\delta$ leads to the following result:

15 $$\begin{aligned} \Psi ={\mathcal {A}}(s, y, z) \partial y+\partial z +{\mathcal {B}}(s, y, z)\partial s+ {\mathcal {C}}(s, y, z, t) \partial \Theta , \end{aligned}$$

where ${\mathcal {A}}(s, y, z),\; {\mathcal {B}}(s, y, z)$ and ${\mathcal {C}}(s, y, z, t)$ are specified functions.

Case 4: We now turn to the fourth condition. In this case, we set $\Upsilon ^{1}=0,\; \Upsilon ^{2}=0,\; \Upsilon ^{4}=0$ and assume $\Upsilon ^{5}\ne 0$, in particular $\Upsilon ^{5}=1$. After performing the necessary simplifications, the governing equations reduce to the following form:

16 $$\begin{aligned} {\left\{ \begin{array}{ll} \Upsilon ^{3}_{\Theta }= \delta _{ \Theta }=0, \\ \Upsilon ^{3}_{x}= \delta _{x}=0, \\ \Upsilon ^{3}_{t}=0, \\ \Upsilon ^{3}_{s}+\Upsilon ^{3}_{y}+\Upsilon ^{3}_{z}=0, \\ \delta _{ts}+\delta _{ty}+\delta _{tz}=0, \\ \delta _{s}+\delta _{y}+\delta _{z}=0. \end{array}\right. } \end{aligned}$$

By solving these governing equations, we obtain the following results: $\Upsilon ^{3}={\mathcal {A}}(s, y, z),\; \Upsilon ^{5}=1,\; \delta ={\mathcal {B}}(s, y, z, t).$ The corresponding symmetry operator in this case is given by:

17 $$\begin{aligned} \Psi = \Upsilon ^{3} \partial y+\Upsilon ^{5}\partial s+ \delta \partial \Theta . \end{aligned}$$

This corresponds to a linear combination of the nonclassical symmetries. Substituting the values of $\Upsilon ^{3},\; \Upsilon ^{5}$ and $\delta$ yields the following expression:

18 $$\begin{aligned} \Psi ={\mathcal {A}}(s, y, z) \partial y+\partial s+ {\mathcal {B}}(s, y, z, t) \partial \Theta , \end{aligned}$$

where ${\mathcal {A}}(s, y, z)$ and ${\mathcal {B}}(s, y, z, t)$ are prescribed functions. For the fourth scenario, the operator appears as a linear combination of nonclassical symmetries.

Case 5: Let us now examine the fifth condition. Here, we set $\Upsilon ^{1}=0,\; \Upsilon ^{2}=0,\; \Upsilon ^{4}=0,\; \Upsilon ^{5}=0$ and assume $\Upsilon ^{3}\ne 0$, specifically taking $\Upsilon ^{3}=1$. After applying the required simplifications, the governing equations reduce to the following form:

19 $$\begin{aligned} & \delta _{\Theta \Theta }=0, \end{aligned}$$

20 $$\begin{aligned} & \delta _{us}+\delta _{uy}+\delta _{uz}=0, \end{aligned}$$

21 $$\begin{aligned} & \beta \delta ^{2}_{u} \delta (1+2\delta )+\beta \delta _{x} (\delta _{x}+3\delta _{u}\delta +\delta _{u})+\alpha (3\delta ^{2}_{xu}+3\delta _{u}\delta _{xxu})+\delta _{xu}(2\beta \delta ^{2}+3\alpha \delta ^{2}_{u})+\beta \delta (\delta _{xx}+\delta _{xu})+\delta _{ut}=0, \end{aligned}$$

22 $$\begin{aligned} & 3\alpha \delta (\delta _{xxus}+\delta _{xxuy}+\delta _{xxuz})+(\delta _{s}+\delta _{y}+\delta _{z})[3\alpha (\delta _{xxu}+\delta _{u}\delta _{xu})+\beta \delta (\delta _{u}+\delta _{s}+\delta _{u}\delta )+\beta \delta _{x}]+\nonumber \\ & (\delta _{us}+\delta _{uy}+\delta _{uz})[a(\delta _{xx}+\delta _{x}\delta _{u}+5\delta _{xu}\delta +\delta ^{2}_{u}\delta )+\beta \delta ^{2}]+\alpha (\delta _{xxxs}+\delta _{xxxy}+\delta _{xxxz})+\nonumber \\ & 3\alpha (\delta _{u}\delta +\delta _{x})(\delta _{xus}+\delta _{xuy}+\delta _{xuz})+(\beta \delta +3\alpha \delta _{xu})(\delta _{xs}+\delta _{xy}+\delta _{xz})+(\delta _{ts}+\delta _{ty}+\delta _{tz})=0. \end{aligned}$$

Solving these governing equations gives the following results: $\Upsilon ^{3}=1,\; \delta ={\mathcal {A}}(s, y, z, t).$ The symmetry operator associated with this case can be written as:

23 $$\begin{aligned} \Psi = \Upsilon ^{3} \partial y+\delta \partial \Theta . \end{aligned}$$

This corresponds to a linear combination of nonclassical symmetries. By substituting the values of $\Upsilon ^{3}$ and $\delta$, the operator takes the following form:

24 $$\begin{aligned} \Psi = \partial y+ {\mathcal {A}}(s, y, z, t) \partial \Theta , \end{aligned}$$

where ${\mathcal {A}}(s, y, z, t)$ is known function.

## Invariant structures via conditional symmetries, determination of auxiliary functions, analytical solutions, and illustrative plots

This part provides a detailed discussion of the conditionally invariant solutions associated with the nonclassical generator derived in the preceding analysis. Here, the unidentified functions are systematically classified, and the corresponding exact solutions are obtained together with their graphical representations.

Case 1: The invariant surface condition in the first formulation is given by:

25 $$\begin{aligned} & \Gamma =\Upsilon ^{1}\frac{\partial \Theta }{\partial t}+ \Upsilon ^{2}\frac{\partial \Theta }{\partial x}+\Upsilon ^{3}\frac{\partial \Theta }{\partial y}+\Upsilon ^{4}\frac{\partial \Theta }{\partial z}+\Upsilon ^{5}\frac{\partial \Theta }{\partial s}-\delta =0. \end{aligned}$$

The result below is obtained by substituting the values of $\Upsilon ^{1},\; \Upsilon ^{2},\; \Upsilon ^{3},\; \Upsilon ^{4},\; \Upsilon ^{5}$ and $\delta$.

26 $$\begin{aligned} \Longrightarrow \Theta _{t} +{\mathcal {A}}(s, y, z)\Theta _{x}+ {\mathcal {B}}(s, y, z) \Theta _{y}+{\mathcal {C}}(s, y, z)\Theta _{z} +{\mathcal {D}}(s, y, z)\Theta _{s}- {\mathcal {E}}(s, y, z, t) =0, \end{aligned}$$

it can now be written as,

27 $$\begin{aligned} \Theta _{t} = {\mathcal {E}}(s, y, z, t)-{\mathcal {A}}(s, y, z)\Theta _{x}- {\mathcal {B}}(s, y, z) \Theta _{y}-{\mathcal {C}}(s, y, z)\Theta _{z} -{\mathcal {D}}(s, y, z)\Theta _{s}, \end{aligned}$$

where ${\mathcal {A}}(s, y, z),\; {\mathcal {B}}(s, y, z),\; {\mathcal {C}}(s, y, z),\; {\mathcal {D}}(s, y, z)$ and ${\mathcal {E}}(s, y, z, t)$ are prescribed functions. Eq. (27) represents the governing PDE, and its solutions are derived by examining distinct cases and assigning particular forms to the unknown functions.

Case 1.1: For this case, the equation takes the form under the assignments ${\mathcal {A}}(s, y, z)=s^{0},\; {\mathcal {B}}(s, y, z)=y^{0},\; {\mathcal {C}}(s, y, z)=z^{0},\; {\mathcal {D}}(s, y, z)=(syz)^{0},\; {\mathcal {E}}(s, y, z, t)=(s+y+z)\sin (t).$

28 $$\begin{aligned} \Theta _{t} = (s+y+z)sin(t)-s^{0}\Theta _{x}- y^{0} \Theta _{y}-z^{0}\Theta _{z} -(syz)^{0}\Theta _{s}, \end{aligned}$$

29 $$\begin{aligned} \Longrightarrow \Theta _{t} = (s+y+z)sin(t)-\Theta _{x}- \Theta _{y}-\Theta _{z} -\Theta _{s}, \end{aligned}$$

The PDE is solved with the aid of Maple software, leading to the following solution involving an arbitrary function.

30 $$\begin{aligned} \Theta (x,y,z,t,s) = 3sin(t)-cos(t)s-cos(t)y-cos(t)z+{\mathcal {H}} (s-x, y-x, z-x, t-x), \end{aligned}$$

where ${\mathcal {H}}(s-x, y-x, z-x, t-x)$ denotes an unspecified function. By considering different scenarios, one can determine explicit forms of this function, with each case producing a distinct form of solution.

Case 1.1.1: Consider the invariant solution in terms of the unknown function ${\mathfrak {h}}(x)$, which can be written as:

31 $$\begin{aligned} \Theta (x,y,z,t,s) = 3sin(t)-cos(t)s-cos(t)y-cos(t)z+ {\mathfrak {h}}(x). \end{aligned}$$

Substituting this invariant solution into Eq. (1) transforms the equation into an ordinary differential equation (ODE).

32 $$\begin{aligned} 3sin(t)-3\beta \left( \frac{d^{2}}{dx^{2}}{\mathfrak {h}}(x)\right) cos(t)=0, \end{aligned}$$

The ODE is solved with the aid of Maple software, yielding the following expression for ${\mathfrak {h}}(x)$:

33 $$\begin{aligned} {\mathfrak {h}}(x)=\frac{x^{2}sin(t)}{2\beta cos(t)}+{\mathscr {E}}_{1}x+{\mathscr {E}}_{2}. \end{aligned}$$

The specific solution is now obtained by combining Eq. (33) with Eq. (31).

34 $$\begin{aligned} \Theta (x,y,z,t,s) = 3sin(t)-cos(t)s-cos(t)y-cos(t)z+ \frac{x^{2}sin(t)}{2\beta cos(t)}+{\mathscr {E}}_{1}x+{\mathscr {E}}_{2}, \end{aligned}$$

that solution is exact solution.

**Fig. 1:**
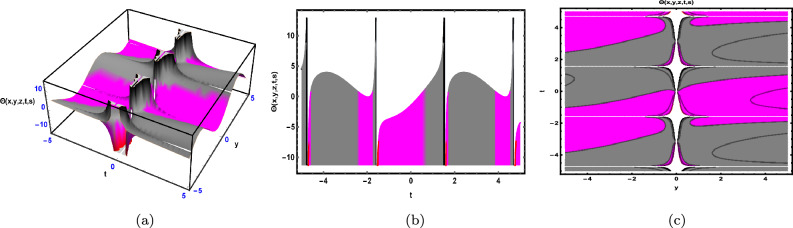
The Solution for Eq. (34) of $\Theta (x,y,z,t,s)$ when ${\mathscr {E}}_{1} =-0.05,\; {\mathscr {E}}_{2} =0.7, \,$ $x = -0.5,\;s = 0.66,\; z = .88,\; \beta = 0.66(y^{2}).$

As depicted in Fig. 1, the graphical visualization of solution (34) for Case 1.1.1 is presented through 3D dynamics (Fig. 1a), 2D dynamics (Fig. 1b), and contour plots (Fig. 1c). The 3D and contour plots are generated over the intervals $-5 \le y \le 5$ and $-5 \le t \le 5$, with the parameters ${\mathscr {E}}_{1},\; {\mathscr {E}}_{2},\; \beta$ and the variables $x,\; s,\; z$ kept fixed. The 2D dynamics (Fig. 1b) is obtained for $-5 \le t \le 5$, where *y* is also fixed.

Case 1.2: Equation (27) takes the following form under the assignments ${\mathcal {A}}(s, y, z)=s^{0},\; {\mathcal {B}}(s, y, z)=y^{0},\; {\mathcal {C}}(s, y, z)=z^{0},\; {\mathcal {D}}(s, y, z)=(syz)^{0},\; {\mathcal {E}}(s, y, z, t)=(s+y+z)\cos (t).$

35 $$\begin{aligned} \Theta _{t} = (s+y+z)cos(t)-s^{0}\Theta _{x}- y^{0} \Theta _{y}-z^{0}\Theta _{z} -(syz)^{0}\Theta _{s}, \end{aligned}$$

36 $$\begin{aligned} \Longrightarrow \Theta _{t} = (s+y+z)cos(t)-\Theta _{x}- \Theta _{y}-\Theta _{z} -\Theta _{s}, \end{aligned}$$

The PDE is solved with the aid of Maple software, which yields the following solution involving an arbitrary function.

37 $$\begin{aligned} \Theta (x,y,z,t,s) = 3cos(t)+sin(t)s+sin(t)y+sin(t)z+{\mathcal {H}} (s-x, y-x, z-x, t-x), \end{aligned}$$

where ${\mathcal {H}}(s-x, y-x, z-x, t-x)$ denotes an undetermined function. Exploring different scenarios makes it possible to evaluate this function, with each case yielding a distinct particular solution.

Case 1.2.1: Suppose the invariant solution involving the unknown function ${\mathfrak {h}}(x)$ is expressed as:

38 $$\begin{aligned} \Theta (x,y,z,t,s) = 3cos(t)+sin(t)s+sin(t)y+sin(t)z+ {\mathfrak {h}}(x). \end{aligned}$$

Substituting this invariant solution into Eq. (1) reduces the equation to ODE.

39 $$\begin{aligned} 3cos(t)+3\beta \left( \frac{d^{2}}{dx^{2}}{\mathfrak {h}}(x)\right) sin(t)=0, \end{aligned}$$

The ODE is solved using Maple software, and the solution for ${\mathfrak {h}}(x)$ is obtained as:

40 $$\begin{aligned} {\mathfrak {h}}(x)=-\frac{x^{2}cos(t)}{2\beta sin(t)}+{\mathscr {E}}_{1}x+{\mathscr {E}}_{2}. \end{aligned}$$

The specific solution is now obtained by combining Eq. (40) with Eq. (38).

41 $$\begin{aligned} \Theta (x,y,z,t,s) = 3cos(t)+sin(t)s+sin(t)y+sin(t)z-\frac{x^{2}cos(t)}{2\beta sin(t)}+{\mathscr {E}}_{1}x+{\mathscr {E}}_{2}, \end{aligned}$$

that solution is exact solution.

**Fig. 2:**
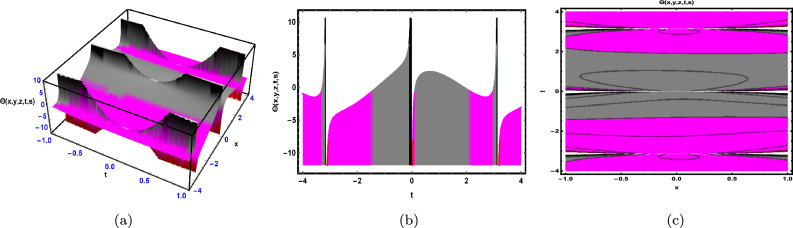
The solution for Eq. (41) of $\Theta (x,y,z,t,s)$ when ${\mathscr {E}}_{1} =-0.23,\; {\mathscr {E}}_{2} =0.13,\;$ $y = -0.25,\;s = 0.5,\; z = .7,\; \beta = 0.6.$

As shown in Fig. 2, the graphical illustration of solution (41) for Case 1.2.1 is presented through 3D dynamics (Fig. 2a), 2D dynamics (Fig. (2b), and contour plots (Fig. 2c). The 3D and contour plots are generated over the intervals $-1 \le x \le 1$ and $-4 \le t \le 4$, with the parameters ${\mathscr {E}}_{1},\; {\mathscr {E}}_{2},\; \beta$ and the variables $y,\; s,\; z$ fixed. The 2D dynamics (Fig. 2b) is obtained for $-4 \le t \le 4$, where *x* is also fixed.

Case 1.3:  The Eq. (27) becomes for ${\mathcal {A}}(s, y, z) \;=\;s^{0},$ ${\mathcal {B}}(s, y, z) \;=\;y,$ ${\mathcal {C}}(s, y, z) \;=\;z,$ ${\mathcal {D}}(s, y, z) \;=\;(y)^{0}$ and ${\mathcal {E}}(s, y, z, t)\;=\;s(cos(t)+sin(t)),$

42 $$\begin{aligned} \Theta _{t} = s(cos(t)+sin(t))-s^{0}\Theta _{x}- y \Theta _{y}-z\Theta _{z} -y^{0}\Theta _{s}, \end{aligned}$$

43 $$\begin{aligned} \Longrightarrow \Theta _{t} = s(cos(t)+sin(t))-\Theta _{x}- y\Theta _{y}-z\Theta _{z} -\Theta _{s}, \end{aligned}$$

The PDE is handled with the aid of Maple software, producing the following solution that involves an arbitrary function.

44 $$\begin{aligned} \Theta (x,y,z,t,s) = s(sin(t)-cos(t))+cos(t)+sin(t)+{\mathcal {H}} (s-x, ye^{-x}, ze^{-x}, t-x), \end{aligned}$$

where ${\mathcal {H}} (s-x, ye^{-x}, ze^{-x}, t-x)$ is an unidentified function. Substituting different situations allows for deriving the value of the unknown function. Each situation leads to a specific particular solution.

Case 1.3.1:  Assume the invariant solution with the unidentified function ${\mathfrak {h}}(x)$ is given by,

45 $$\begin{aligned} \Theta (x,y,z,t,s) = s(sin(t)-cos(t))+cos(t)+sin(t)+ {\mathfrak {h}}(x). \end{aligned}$$

By substituting this invariant solution into Eq. (1) and the result is obtained in the form of ODE.

46 $$\begin{aligned} cos(t)+sin(t)+\beta \left( \frac{d^{2}}{dx^{2}}{\mathfrak {h}}(x)\right) sin(t)-\beta \left( \frac{d^{2}}{dx^{2}}{\mathfrak {h}}(x)\right) cos(t)=0, \end{aligned}$$

The ODE is solved using Maple software, and the resulting solution for ${\mathfrak {h}}(x)$ is given by:

47 $$\begin{aligned} {\mathfrak {h}}(x)=\frac{x^{2}(cos(t)+sin(t))}{2\beta (cos(t)-sin(t))}+{\mathscr {E}}_{1}x+{\mathscr {E}}_{2}. \end{aligned}$$

The specific solution is now obtained by combining Eq. (47) with Eq. (45).

48 $$\begin{aligned} \Theta (x,y,z,t,s) = s(sin(t)-cos(t))+cos(t)+sin(t)+ \frac{x^{2}(cos(t)+sin(t))}{2\beta (cos(t)-sin(t))}+{\mathscr {E}}_{1}x+{\mathscr {E}}_{2}, \end{aligned}$$

that solution is exact solution.

**Fig. 3:**
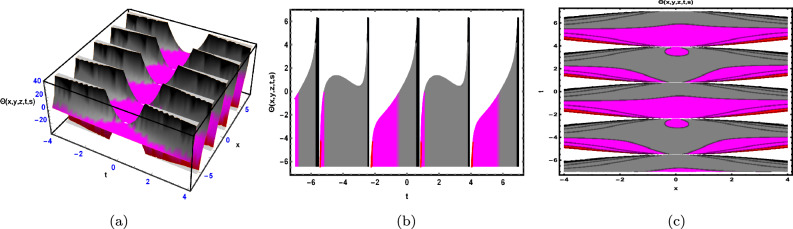
The solution for Eq. (48) of $\Theta (x,y,z,t,s)$ when ${\mathscr {E}}_{1} = -0.2,\; {\mathscr {E}}_{2} = 0.3,\;$ $s = 0.66,\; z = 0.77,\; \beta = 0.33.$

As shown in Fig. 3, the graphical representation of the solution (48) for case 1.3.1 is depicted by using 3D dynamics (Fig. 3a), 2D dynamics (Fig. 3b) and Contour plots dynamics (Fig. 3c). Graphical representation 3D dynamics (Fig. 3a) and Contour plots dynamics (3(c)) are obtained by using the intervals $-4 \le x \le 4$ and $-7 \le t \le 7,$ in these structures the parameters ${\mathscr {E}}_{1},\; {\mathscr {E}}_{2},\; \beta$ and variables $y,\; s,\; z$ are fixed. While 2D dynamics (3(b)) is obtained by using the interval $-7 \le t \le 7,$ in this structure *x* is also fixed.

Case 2: The condition for the invariant surface in the second condition formulation is,

49 $$\begin{aligned} & \Gamma = \Upsilon ^{2}\frac{\partial \Theta }{\partial x}+\Upsilon ^{3}\frac{\partial \Theta }{\partial y}+\Upsilon ^{4}\frac{\partial \Theta }{\partial z}+\Upsilon ^{5}\frac{\partial \Theta }{\partial s}-\delta =0. \end{aligned}$$

The following result is obtained by substituting the values of $\Upsilon ^{2},\; \Upsilon ^{3},\; \Upsilon ^{4},\; \Upsilon ^{5}$ and $\delta$.

50 $$\begin{aligned} \Longrightarrow \Theta _{x}+ {\mathcal {A}}(s, y, z) \Theta _{y}+{\mathcal {B}}(s, y, z)\Theta _{z} +{\mathcal {C}}(s, y, z)\Theta _{s}- {\mathcal {D}}(s, y, z, t) =0, \end{aligned}$$

it can now be written as,

51 $$\begin{aligned} \Theta _{x} = {\mathcal {D}}(s, y, z, t)- {\mathcal {A}}(s, y, z) \Theta _{y}-{\mathcal {B}}(s, y, z)\Theta _{z} -{\mathcal {C}}(s, y, z)\Theta _{s}, \end{aligned}$$

here ${\mathcal {A}}(s, y, z),\; {\mathcal {B}}(s, y, z),\; {\mathcal {C}}(s, y, z)$ and ${\mathcal {D}}(s, y, z, t)$ are prescribed functions. Eq. (51) denotes the governing PDE, and its solutions are obtained by considering different cases and assigning specific forms to the unknown functions.

Case 2.1:  The above equation becomes for ${\mathcal {A}}(s, y, z) \;=\;y,$ ${\mathcal {B}}(s, y, z) \;=\;y^{0},$ ${\mathcal {C}}(s, y, z) \;=\;s$ and ${\mathcal {D}}(s, y, z, t)\;=\;(s+y+z)t,$

52 $$\begin{aligned} \Theta _{x} = (s+y+z)t- y \Theta _{y}-z^{0}\Theta _{z} -s\Theta _{s}, \end{aligned}$$

53 $$\begin{aligned} \Longrightarrow \Theta _{x} = (s+y+z)t- y\Theta _{y}-\Theta _{z} -s\Theta _{s}, \end{aligned}$$

The PDE is solved with the assistance of Maple software, resulting in the following solution that involves an arbitrary function.

54 $$\begin{aligned} \Theta (x,y,z,t,s) =t(xz+s+y-\frac{x^{2}}{2})+{\mathcal {H}} (se^{-x}, ye^{-x}, z-x, t), \end{aligned}$$

where ${\mathcal {H}}(se^{-x},\, ye^{-x},\, z-x,\, t)$ denotes an undetermined function. By considering different scenarios, one can determine explicit forms of this function, with each case yielding a distinct solution.

Case 2.1.1:  Consider the invariant solution involving the unknown function ${\mathfrak {h}}(x)$, which is expressed as:

55 $$\begin{aligned} \Theta (x,y,z,t,s) = t(xz+s+y-\frac{x^{2}}{2})+ {\mathfrak {h}}(x). \end{aligned}$$

By substituting this invariant solution into Eq. (1) and the result is obtained in the form of ODE.

56 $$\begin{aligned} 2+x+\beta t^{2}(z-2x-2)-\beta t\left( \frac{d}{dx}{\mathfrak {h}}(x)\right) =0, \end{aligned}$$

The ODE is solved with the aid of Maple software, yielding the following solution for ${\mathfrak {h}}(x)$:

57 $$\begin{aligned} {\mathfrak {h}}(x)=\frac{2+x+\beta t^{2}(z-2x-2)}{\beta t}+{\mathscr {E}}_{1}. \end{aligned}$$

The specific solution is now obtained by combining Eq. (57) with Eq. (55).

58 $$\begin{aligned} \Theta (x,y,z,t,s) = t(xz+s+y-\frac{x^{2}}{2})+\frac{2+x+\beta t^{2}(z-2x-2)}{\beta t}+{\mathscr {E}}_{1}, \end{aligned}$$

that solution is exact solution.

**Fig. 4:**
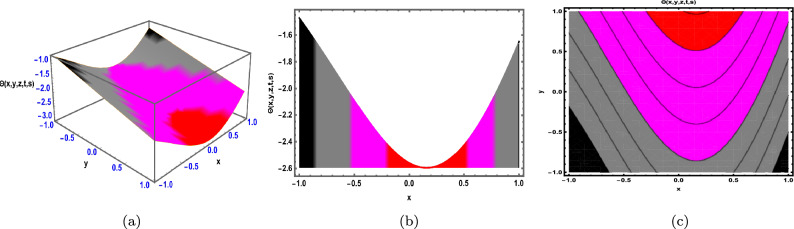
The Solution for Eq. (58) of $\Theta (x,y,z,t,s)$ when ${\mathscr {E}}_{1} =-0.62,\; t = -0.55,\; z = 0.95,\;$ $s = 0.61,\; \beta = 1.77.$

As demonstrated in Fig. 4, the graphical representation of solution (58) for Case 2.1.1 is presented through 3D dynamics (Fig. (4a), 2D dynamics (Fig. 4b), and contour plots (Fig. 4c). The 3D and contour plots are generated over the intervals $-1 \le x \le 1$ and $-1 \le y \le 1$, with the parameters ${\mathscr {E}}_{1},\; {\mathscr {E}}_{2},\; \beta$ and the variables $t,\; s,\; z$ fixed. The 2D dynamics (Fig. 4b) is obtained for $-1 \le x \le 1$, where *y* is also fixed.

Case 2.2:  The Eq. (51) becomes for ${\mathcal {A}}(s, y, z) \;=\;y+z,$ ${\mathcal {B}}(s, y, z) \;=\;s+z,$ ${\mathcal {C}}(s, y, z) \;=\;z^{0}$ and ${\mathcal {D}}(s, y, z, t)\;=\;(s+y+z)e^{t},$

59 $$\begin{aligned} \Theta _{x} = (s+y+z)e^{t}- (y+z) \Theta _{y}-(z+s)\Theta _{z} -z^{0}\Theta _{s}, \end{aligned}$$

60 $$\begin{aligned} \Longrightarrow \Theta _{x} = (s+y+z)e^{t}- (y+z) \Theta _{y}-(z+s)\Theta _{z} -\Theta _{s}, \end{aligned}$$

The PDE is solved with the aid of Maple software, resulting in the following solution involving an arbitrary function.

61 $$\begin{aligned} \Theta (x,y,z,t,s) = e^{t}[x(1+s)-(2+s)+y-\frac{x^{2}}{2}]+{\mathcal {H}} (t, s-x, (z+1+s)e^{-x}, (y-2-s-x(z+1+s))e^{-x}), \end{aligned}$$

where ${\mathcal {H}}(t,\, s-x,\, (z+1+s)e^{-x},\, (y-2-s-x(z+1+s))e^{-x})$ represents an undetermined function. By considering alternative scenarios, explicit forms of this function can be obtained, and each case results in a distinct particular solution.

Case 2.2.1:  Consider the invariant solution with the unidentified function ${\mathfrak {h}}(x)$ is given by,

62 $$\begin{aligned} \Theta (x,y,z,t,s) = e^{t}[x(1+s)-(2+s)+y-\frac{x^{2}}{2}]+ {\mathfrak {h}}(x). \end{aligned}$$

By substituting this invariant solution into Eq. (1) and the result is obtained in the form of ODE.

63 $$\begin{aligned} e^{t}x+\beta ( e^{t})^{2}(1+s-2x)+\beta e^{t} \left( \frac{d}{dx}{\mathfrak {h}}(x)\right) +\beta e^{t}\left( \frac{d^{2}}{dx^{2}}{\mathfrak {h}}(x)\right) x =0, \end{aligned}$$

The ODE is solved using Maple software, and the solution for ${\mathfrak {h}}(x)$ is obtained as:

64 $$\begin{aligned} {\mathfrak {h}}(x)=\frac{x^{2}e^{t}}{2}-(1+s)xe^{t}-\frac{x^{2}}{4\beta }+{\mathscr {E}}_{1}ln(x)+{\mathscr {E}}_{2}. \end{aligned}$$

The specific solution is now obtained by combining Eq. (64) with Eq. (62).

65 $$\begin{aligned} \Theta (x,y,z,t,s) =e^{t}[y-(2+s)]-\frac{x^{2}}{4\beta }+{\mathscr {E}}_{1}ln(x)+{\mathscr {E}}_{2}, \end{aligned}$$

that solution is exact solution.

**Fig. 5:**
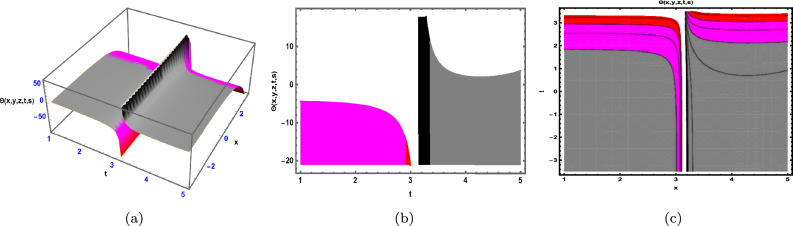
The solution for Eq. (65) of $\Theta (x,y,z,t,s)$ when ${\mathscr {E}}_{1} = 0.2,\; {\mathscr {E}}_{2} =0.35,\;$ $y = -0.5,\; z = 0.77,\; s = 0.66,\; \beta = 0.88.$

As signified in Fig. 5, the graphical depiction of solution (65) for Case 2.2.1 is presented through 3D dynamics (Fig. 5a), 2D dynamics (Fig. 5b), and contour plots (Fig. 5c). The 3D and contour plots are generated over the intervals $1 \le x \le 5$ and $-3.5 \le t \le 3.5$, with the parameters ${\mathscr {E}}_{1},\; {\mathscr {E}}_{2},\; \beta$ and the variables $y,\; s,\; z$ fixed. The 2D dynamics (Fig. 5b) is obtained for $1 \le t \le 5$, where *x* is also fixed.

Case 2.3:  The Eq. (51) becomes for ${\mathcal {A}}(s, y, z) \;=\;s^{0},$ ${\mathcal {B}}(s, y, z) \;=\;s+z,$ ${\mathcal {C}}(s, y, z) \;=\;z^{0}$ and ${\mathcal {D}}(s, y, z, t)\;=\;ycos(t),$

66 $$\begin{aligned} \Theta _{x} = ycos(t)- s^{0} \Theta _{y}-(s+z)\Theta _{z} -z^{0}\Theta _{s}, \end{aligned}$$

67 $$\begin{aligned} \Longrightarrow \Theta _{x} =ycos(t)- \Theta _{y}-(s+z)\Theta _{z} -\Theta _{s}, \end{aligned}$$

The PDE is solved with the assistance of Maple software, leading to the following solution that involves an undetermined function.

68 $$\begin{aligned} \Theta (x,y,z,t,s) =- \frac{x^{2}cos(t)}{2}+cos(t)xy+{\mathcal {H}} (s-x, t, (z+1+s)e^{-x}, y-x), \end{aligned}$$

where ${\mathcal {H}}(s-x, t, (z+1+s)e^{-x}, y-x)$ represents an undetermined function.

By examining different scenarios, explicit forms of this function can be obtained, with each case yielding a distinct particular solution.

Case 2.3.1:  Assume that the invariant solution involving the unknown function ${\mathfrak {h}}(x)$ is expressed as:

69 $$\begin{aligned} \Theta (x,y,z,t,s) = - \frac{x^{2}cos(t)}{2}+cos(t)xy+ {\mathfrak {h}}(x). \end{aligned}$$

By substituting this invariant solution into Eq. (1) and the result is obtained in the form of ODE.

70 $$\begin{aligned} \beta cos(t)^{2}(y-2x)-sin(t)x+\beta cos(t)\left( \frac{d}{dx}{\mathfrak {h}}(x)\right) -\beta xcos(t)\left( \frac{d^{2}}{dx^{2}}{\mathfrak {h}}(x)\right) =0, \end{aligned}$$

The ODE is solved with the help of Maple software, and the solution for ${\mathfrak {h}}(x)$ is obtained as:

71 $$\begin{aligned} {\mathfrak {h}}(x)=\frac{x^{2}sin(t)}{4\beta cos(t)} + \frac{x^{2}cos(t)}{2}-cos(t)xy+{\mathscr {E}}_{1}ln(x)+{\mathscr {E}}_{2}. \end{aligned}$$

The specific solution is now obtained by combining Eq. (71) with Eq. (69).

72 $$\begin{aligned} \Theta (x,y,z,t,s) = \frac{x^{2}sin(t)}{4\beta cos(t)}+{\mathscr {E}}_{1}ln(x)+{\mathscr {E}}_{2}, \end{aligned}$$

that solution is exact solution.

**Fig. 6:**
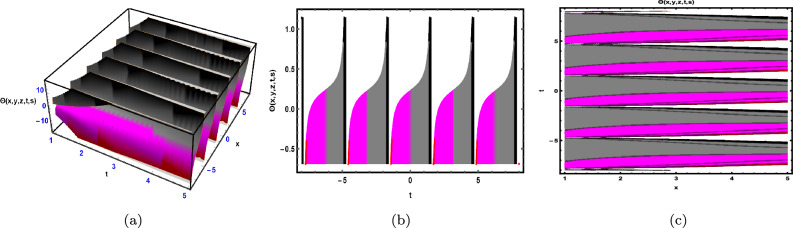
The solution for Eq. (72) of $\Theta (x,y,z,t,s)$ when ${\mathscr {E}}_{1} = 0.2,\;$ ${\mathscr {E}}_{2} =0.3,\; \beta = 0.89.$

As represent in Fig. 6, the graphical illustration of solution (72) for Case 2.3.1 is presented through 3D dynamics (Fig. 6a), 2D dynamics (Fig. 6b), and contour plots (Fig. 6c). The 3D and contour plots are generated over the intervals $1 \le x \le 5$ and $-8 \le t \le 8$, with the parameters ${\mathscr {E}}_{1},\; {\mathscr {E}}_{2},\; \beta$ and the variables $y,\; s,\; z$ fixed. The 2D dynamics (Fig. 6b) is obtained for $-8 \le t \le 8$, where *x* is also fixed.

Case 3: The condition for the invariant surface in the second condition formulation is,

73 $$\begin{aligned} & \Gamma = \Upsilon ^{3}\frac{\partial \Theta }{\partial y}+\Upsilon ^{4}\frac{\partial \Theta }{\partial z}+\Upsilon ^{5}\frac{\partial \Theta }{\partial s}-\delta =0. \end{aligned}$$

The following result is obtained by substituting the values of $\Upsilon ^{3},\; \Upsilon ^{4},\; \Upsilon ^{5}$ and $\delta$.

74 $$\begin{aligned} \Longrightarrow {\mathcal {A}}(s, y, z) \Theta _{y}+\Theta _{z} +{\mathcal {B}}(s, y, z)\Theta _{s}- {\mathcal {C}}(s, y, z, t) =0, \end{aligned}$$

it can now be written as,

75 $$\begin{aligned} \Theta _{z} = \gamma (s, y, z, t)- {\mathcal {A}}(s, y, z) \Theta _{y} -{\mathcal {B}}(s, y, z)\Theta _{s}, \end{aligned}$$

where ${\mathcal {A}}(s, y, z),\; {\mathcal {B}}(s, y, z)$ and ${\mathcal {C}}(s, y, z, t)$ are prescribed functions. Eq. (75) denotes the governing PDE, and its solutions are obtained by considering distinct cases and assigning suitable forms to the unknown functions.

Case 3.1:  The above equation becomes for ${\mathcal {A}}(s, y, z) \;=\;y^{0},$ ${\mathcal {B}}(s, y, z) \;=\;s^{0}$ and ${\mathcal {C}}(s, y, z, t)\;=\;(y+z)cosh(t),$

76 $$\begin{aligned} \Theta _{z} = (y+z)cosh(t)- y^{0} \Theta _{y} -s^{0}\Theta _{s}, \end{aligned}$$

77 $$\begin{aligned} \Longrightarrow \Theta _{z} = (y+z)cosh(t)- \Theta _{y} -\Theta _{s}, \end{aligned}$$

The PDE is solved using Mapple software, yielding the following solution with an unknown function.

78 $$\begin{aligned} \Theta (x,y,z,t,s) =yzcosh(t)+{\mathcal {H}} (x, s-y, z-y, t), \end{aligned}$$

where ${\mathcal {H}}(x, s-y, z-y, t)$ represents an undetermined function. By exploring different scenarios, one can determine explicit forms of this function, with each case producing a distinct particular solution.

Case 3.1.1: Assume that the invariant solution involving the unknown function ${\mathfrak {h}}(x)$ is expressed as:

79 $$\begin{aligned} \Theta (x,y,z,t,s) = yzcosh(t)+ {\mathfrak {h}}(x). \end{aligned}$$

By substituting this invariant solution into Eq. (1) and the result is obtained in the form of ODE.

80 $$\begin{aligned} sinh(t)(y+z)+\beta cosh(t)\left( \frac{d^{2}}{dx^{2}}{\mathfrak {h}}(x)\right) (y+z)=0, \end{aligned}$$

The ODE is solved using Maple software, and the solution for ${\mathfrak {h}}(x)$ is obtained as:

81 $$\begin{aligned} {\mathfrak {h}}(x)=-\frac{x^{2}sinh(t)}{2\beta cosh(t)}+{\mathscr {E}}_{1}ln(x)+{\mathscr {E}}_{2}. \end{aligned}$$

The specific solution is now obtained by combining Eq. (81) with Eq. (79).

82 $$\begin{aligned} \Theta (x,y,z,t,s) = yzcosh(t)-\frac{x^{2}sinh(t)}{2\beta cosh(t)}+{\mathscr {E}}_{1}ln(x)+{\mathscr {E}}_{2}, \end{aligned}$$

that solution is exact solution.

**Fig. 7:**
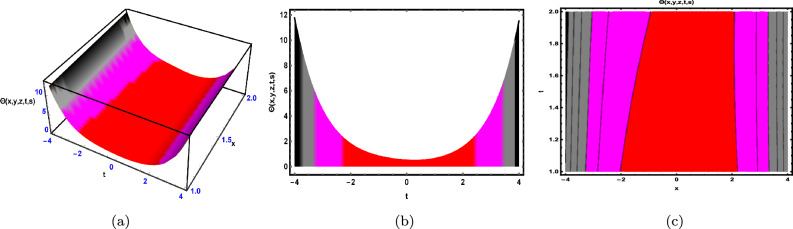
The solution for Eq. (82) of $$\Theta (x,y,z,t,s)$$ when $${\mathscr {E}}_{1} = 1,\; {\mathscr {E}}_{2} =0.2,\; y = 0.55,\;$$$$z = 0.77,\; \beta = 3.$$

As shown in Fig. 7, the graphical representation of solution (82) for Case 3.1.1 is presented through 3D dynamics (Fig. 7a), 2D dynamics (Fig. 7b), and contour plots (Fig. 7c). The 3D and contour plots are generated over the intervals $1 \le x \le 2$ and $-4 \le t \le 4$, with the parameters ${\mathscr {E}}_{1},\; {\mathscr {E}}_{2},\; \beta$ and the variables $y,\; s,\; z$ fixed. The 2D dynamics (Fig. 7b) is obtained for $-4 \le t \le 4$, where *x* is also fixed.

Case 3.2:  The Eq. (75) becomes for ${\mathcal {A}}(s, y, z) \;=\;y,$ ${\mathcal {B}}(s, y, z) \;=\;s^{0}$ and ${\mathcal {C}}(s, y, z, t)\;=\;(s+z)sinh(t),$

83 $$\begin{aligned} \Theta _{z} = (s+z)sinh(t)- y\Theta _{y} -s^{0}\Theta _{s}, \end{aligned}$$

84 $$\begin{aligned} \Longrightarrow \Theta _{z} = (s+z)sinh(t)- y\Theta _{y} -\Theta _{s}, \end{aligned}$$

The PDE is solved with the aid of Maple software, leading to the following solution that involves an arbitrary function.

85 $$\begin{aligned} \Theta (x,y,z,t,s) =sinh(t)ln(t)(z+s-ln(t)) +{\mathcal {H}} (t, x, z-ln(y), z-ln(y)), \end{aligned}$$

where ${\mathcal {H}}(t, x, z-\ln (y), z-\ln (y))$ represents an undetermined function. By examining different scenarios, explicit forms of this function can be obtained, with each case yielding a distinct solution.

Case 3.2.1: Suppose the invariant solution associated with the unknown function ${\mathfrak {h}}(x)$ is represented as:

86 $$\begin{aligned} \Theta (x,y,z,t,s) = sinh(t)ln(t)(z+s-ln(t))+ {\mathfrak {h}}(x). \end{aligned}$$

By substituting this invariant solution into Eq. (1) and the result is obtained in the form of ODE.

87 $$\begin{aligned} \frac{(z+s-2ln(y)+2yln(y))}{y}\left( \frac{d^{2}}{dx^{2}}{\mathfrak {h}}(x)\right) (sinh(t)+cosh(t)) =0, \end{aligned}$$

The ODE is solved with the aid of Maple software, yielding the following solution for ${\mathfrak {h}}(x)$:

88 $$\begin{aligned} {\mathfrak {h}}(x)=-\frac{x^{2}cosh(t)}{2\beta sinh(t)}+{\mathscr {E}}_{1}ln(x)+{\mathscr {E}}_{2}. \end{aligned}$$

The specific solution is now obtained by combining Eq. (88) with Eq. (86).

89 $$\begin{aligned} \Theta (x,y,z,t,s) =sinh(t)ln(t)(z+s-ln(t))-\frac{x^{2}cosh(t)}{2\beta sinh(t)}+{\mathscr {E}}_{1}ln(x)+{\mathscr {E}}_{2}, \end{aligned}$$

that solution is exact solution.

**Fig. 8:**
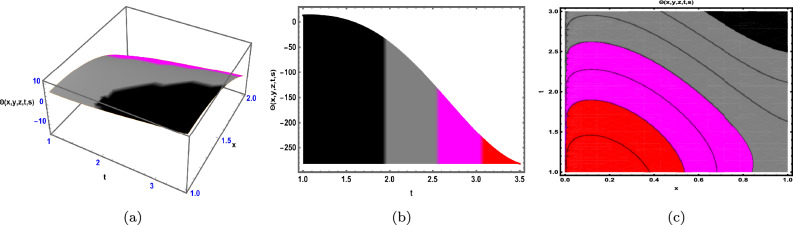
The solution for Eq. (89) of $$\Theta (x,y,z,t,s)$$ when $${\mathscr {E}}_{1} = -0.2,\; {\mathscr {E}}_{2} =0.55,\;$$$$s = 0.61,\; z = -0.95,\; \beta = -0.77.$$

As illustrated in Fig. 8, the graphical representation of solution (89) for Case 3.2.1 is presented through 3D dynamics (Fig. 8a), 2D dynamics (Fig. 8b), and contour plots (Fig. 8c). The 3D and contour plots are generated over the intervals $1 \le x \le 2$ and $1 \le t \le 3.5$, with the parameters ${\mathscr {E}}_{1},\; {\mathscr {E}}_{2},\; \beta$ and the variables $y,\; s,\; z$ fixed. The 2D dynamics (Fig. 8b) is obtained for $1 \le t \le 3.5$, where *x* is also fixed.

Case 3.3:  The Eq. (75) becomes for ${\mathcal {A}}(s, y, z) \;=\;y^{0},$ ${\mathcal {B}}(s, y, z) \;=\;s^{0}$ and ${\mathcal {C}}(s, y, z, t)\;=\;t(cosh(y)+sinh(s)+cosh(z)),$

90 $$\begin{aligned} \Theta _{z} =t(cosh(y)+sinh(s)+cosh(z))- y^{0} \Theta _{y} -s^{0}\Theta _{s}, \end{aligned}$$

91 $$\begin{aligned} \Longrightarrow \Theta _{z} =t(cosh(y)+sinh(s)+cosh(z))- \Theta _{y} -\Theta _{s}, \end{aligned}$$

The PDE is solved with the help of Maple software, resulting in the following solution that contains an arbitrary function.

92 $$\begin{aligned} \Theta (x,y,z,t,s) =t(sinh(y)+cosh(s)+sinh(z))+{\mathcal {H}} (s-y, t, z-y, x), \end{aligned}$$

where ${\mathcal {H}}(s-y, t, z-y, x)$ denotes an undetermined function. By examining different scenarios, one can obtain explicit forms of this function, with each case producing a distinct solution.

Case 3.3.1:  Consider the invariant solution involving the unknown function ${\mathfrak {h}}(x)$, which can be written as:

93 $$\begin{aligned} \Theta (x,y,z,t,s) = t(sinh(y)+cosh(s)+sinh(z))+ {\mathfrak {h}}(x). \end{aligned}$$

By substituting this invariant solution into Eq. (1) and the result is obtained in the form of ODE.

94 $$\begin{aligned} cosh(y)+sinh(s)+cosh(z)+\beta t(cosh(y)+sinh(s)+cosh(z))\left( \frac{d^{2}}{dx^{2}}{\mathfrak {h}}(x)\right) =0, \end{aligned}$$

The ODE is solved using Maple software, and the resulting expression for ${\mathfrak {h}}(x)$ is obtained as:

95 $$\begin{aligned} {\mathfrak {h}}(x)=-\frac{x^{2}}{2\beta t} +{\mathscr {E}}_{1}x+{\mathscr {E}}_{2}. \end{aligned}$$

The specific solution is now obtained by combining Eq. (95) with Eq. (93).

96 $$\begin{aligned} \Theta (x,y,z,t,s) = t(sinh(y)+cosh(s)+sinh(z)) -\frac{x^{2}}{2\beta t} +{\mathscr {E}}_{1}x+{\mathscr {E}}_{2}, \end{aligned}$$

that solution is exact solution.

**Fig. 9:**
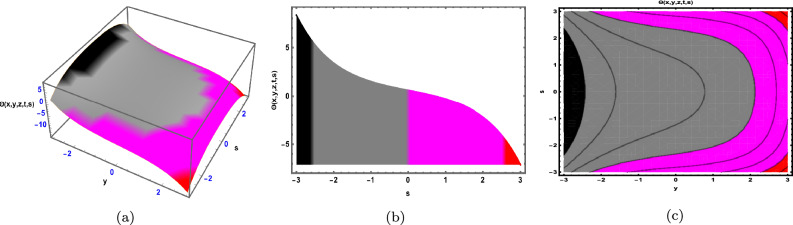
The solution for Eq. (96) of $\Theta (x,y,z,t,s)$ when ${\mathscr {E}}_{1} = -0.2,\; {\mathscr {E}}_{2} =0.55,\; x = -0.61,\;$ $t = -0.77,\; z = -0.95,\; \beta = -2.77.$

As illustrated in Fig. 9, the graphical representation of solution (96) for Case 3.3.1 is presented through 3D dynamics (Fig. (9a), 2D dynamics (Fig. 9b), and contour plots (Fig. 9c). The 3D dynamics (Fig. 9a) and contour plots (Fig. 9c) are generated over the intervals $-3 \le y \le 3$ and $-3 \le s \le 3$, with the parameters ${\mathscr {E}}_{1},\; {\mathscr {E}}_{2},\; \beta$ and the variables $x,\; t,\; z$ fixed. The 2D dynamics (Fig. 9b) is obtained for $-3 \le s \le 3$, where *y* is also fixed.

Case 4: The condition for the invariant surface in the forth condition formulation is,

97 $$\begin{aligned} & \Gamma = \Upsilon ^{3}\frac{\partial \Theta }{\partial y}+\Upsilon ^{5}\frac{\partial \Theta }{\partial s}-\delta =0. \end{aligned}$$

Now, the following outcome may be achieved by altering the values of $\Upsilon ^{3},\ \Upsilon ^{5}$ and $\delta$.

98 $$\begin{aligned} \Longrightarrow {\mathcal {A}}(s, y, z) \Theta _{y} +\Theta _{s}- {\mathcal {B}}(s, y, z, t) =0, \end{aligned}$$

it can now be written as,

99 $$\begin{aligned} \Theta _{s} = {\mathcal {B}}(s, y, z, t)- {\mathcal {A}}(s, y, z) \Theta _{y}, \end{aligned}$$

where ${\mathcal {A}}(s, y, z)$ and ${\mathcal {B}}(s, y, z, t)$ are prescribed functions. Eq. (99) denotes the governing PDE, and its solutions are obtained by considering distinct cases and assigning suitable forms to the unknown functions.

Case 4.1:  The above equation becomes for ${\mathcal {A}}(s, y, z) \;=\;y^{0}$ and ${\mathcal {B}}(s, y, z, t)\;=\;(s+z)cos(t),$

100 $$\begin{aligned} \Theta _{s} = (s+z)cos(t)- y^{0} \Theta _{y}, \end{aligned}$$

101 $$\begin{aligned} \Longrightarrow \Theta _{s} = (s+z)cos(t)- \Theta _{y}, \end{aligned}$$

The PDE is solved with the aid of Maple software, resulting in the following solution involving an undetermined function.

102 $$\begin{aligned} \Theta (x,y,z,t,s) =ycos(t)(s+z)-\frac{y^{2}cost}{2}+{\mathcal {H}} (x, s-y, z, t), \end{aligned}$$

where ${\mathcal {H}}(x, s-y, z, t)$ denotes an undetermined function. By examining different scenarios, explicit forms of this function can be determined, with each case yielding a distinct particular solution.

Case 4.1.1: Assume that the invariant solution involving the unknown function ${\mathfrak {h}}(x)$ is expressed as:

103 $$\begin{aligned} \Theta (x,y,z,t,s) = ycos(t)(s+z)-\frac{y^{2}cost}{2}+ {\mathfrak {h}}(x). \end{aligned}$$

Substituting this invariant solution into Eq. (1) reduces the equation to an ODE.

104 $$\begin{aligned} \beta cos(t)\left( \frac{d^{2}}{dx^{2}}{\mathfrak {h}}(x)\right) (s+y+z)-sin(t)(s+y+z)=0, \end{aligned}$$

The ODE is solved using Maple software, and the solution for ${\mathfrak {h}}(x)$ is obtained as:

105 $$\begin{aligned} {\mathfrak {h}}(x)=\frac{x^{2}sin(t)}{2\beta cos(t)}+{\mathscr {E}}_{1}x+{\mathscr {E}}_{2}. \end{aligned}$$

The specific solution is now obtained by combining Eq. (105) with Eq. (103).

106 $$\begin{aligned} \Theta (x,y,z,t,s) = ycos(t)(s+z)-\frac{y^{2}cost}{2}+\frac{x^{2}sin(t)}{2\beta cos(t)}+{\mathscr {E}}_{1}x+{\mathscr {E}}_{2}, \end{aligned}$$

that solution is exact solution.

**Fig. 10:**
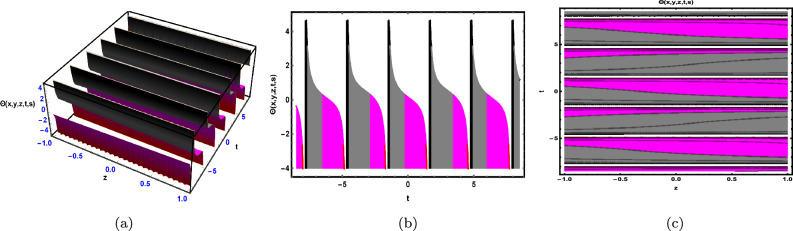
The solution for Eq. (106) of $$\Theta (x,y,z,t,s)$$ when $${\mathscr {E}}_{1} = -0.2,\; {\mathscr {E}}_{2} =0.5,\; x = 0.95,\;$$$$y = -0.55,\; s = 0.61,\; \beta = -0.77.$$

As signified in Fig. 10, the graphical representation of solution (106) for Case 4.1.1 is presented through 3D dynamics (Fig. 10a), 2D dynamics (Fig. 10b), and contour plots (Fig. 10c). The 3D dynamics (Fig. 10a) and contour plots (Fig. 10c) are generated over the intervals $-1 \le z \le 1$ and $-8.5 \le t \le 8.5$, with the parameters ${\mathscr {E}}_{1},\; {\mathscr {E}}_{2},\; \beta$ and the variables $x,\; y,\; s$ fixed. The 2D dynamics (Fig. 10b) is obtained for $-8.5 \le t \le 8.5$, where *z* is also fixed.

Case 4.2:  The Eq. (99) becomes for ${\mathcal {A}}(s, y, z) \;=\;y^{0}$ and ${\mathcal {B}}(s, y, z, t)\;=\;(s+z)sin(t),$

107 $$\begin{aligned} \Theta _{s} = (s+z)sin(t)- y^{0} \Theta _{y}, \end{aligned}$$

108 $$\begin{aligned} \Longrightarrow \Theta _{s} = (s+z)sin(t)- \Theta _{y}, \end{aligned}$$

The PDE is solved with the help of Maple software, leading to the following solution that involves an unspecified function.

109 $$\begin{aligned} \Theta (x,y,z,t,s) =ysin(t)(s+z)-\frac{y^{2}sint}{2}+{\mathcal {H}} (x, s-y, z, t), \end{aligned}$$

where ${\mathcal {H}}(x, s-y, z, t)$ denotes an undetermined function. By exploring different scenarios, explicit forms of this function can be obtained, with each case yielding a distinct solution.

Case 4.2.1: Suppose the invariant solution associated with the unknown function ${\mathfrak {h}}(x)$ is written as:

110 $$\begin{aligned} \Theta (x,y,z,t,s) = ysin(t)(s+z)-\frac{y^{2}sint}{2}+ {\mathfrak {h}}(x). \end{aligned}$$

By substituting this invariant solution into Eq. (1) and the result is obtained in the form of ODE.

111 $$\begin{aligned} \beta sin(t)\left( \frac{d^{2}}{dx^{2}}{\mathfrak {h}}(x)\right) (s+y+z)-cos(t)(s+y+z)=0, \end{aligned}$$

The ODE is solved with the aid of Maple software, yielding the following solution for ${\mathfrak {h}}(x)$:

112 $$\begin{aligned} {\mathfrak {h}}(x)=-\frac{x^{2}cos(t)}{2\beta sin(t)}+{\mathscr {E}}_{1}x+{\mathscr {E}}_{2}. \end{aligned}$$

The specific solution is now obtained by combining Eq. (112) with Eq. (110).

113 $$\begin{aligned} \Theta (x,y,z,t,s) = ysin(t)(s+z)-\frac{y^{2}sint}{2}-\frac{x^{2}cos(t)}{2\beta sin(t)}+{\mathscr {E}}_{1}x+{\mathscr {E}}_{2}, \end{aligned}$$

that solution is exact solution.

**Fig. 11:**
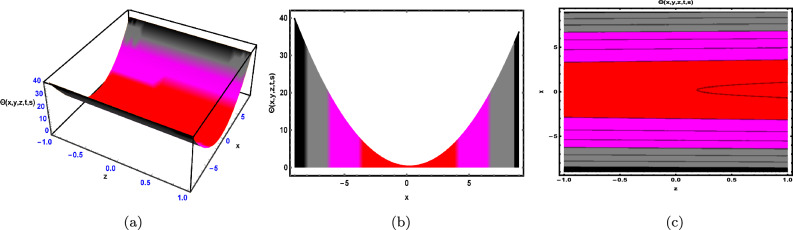
The solution for Eq. (113) of $$\Theta (x,y,z,t,s)$$ when $${\mathscr {E}}_{1} = -0.2,\; {\mathscr {E}}_{2} =0.5,\;$$$$t = 0.95,\; y = -0.55,\; s = 0.61,\; \beta = -0.77.$$

As demonstrated in Fig. 11, the graphical representation of solution (113) for Case 4.2.1 is presented through 3D dynamics (Fig. 11a), 2D dynamics (Fig. 11b), and contour plots (Fig. 11c). The 3D dynamics (Fig. 11a) and contour plots (Fig. 11c) are generated over the intervals $-1 \le z \le 1$ and $-9 \le x \le 9$, with the parameters ${\mathscr {E}}_{1},\; {\mathscr {E}}_{2},\; \beta$ and the variables $t,\; y,\; s$ fixed. The 2D dynamics (Fig. 11b) is obtained for $-9 \le x \le 9$, where *z* is also fixed.

Case 4.3:  The Eq. (99) becomes for ${\mathcal {A}}(s, y, z) \;=\;y^{0}$ and ${\mathcal {B}}(s, y, z, t)\;=\;(sin(s)+cos(y)+sin(z))e^{t},$

114 $$\begin{aligned} \Theta _{s} = (sin(s)+cos(y)+sin(z))e^{t}- y^{0} \Theta _{y}, \end{aligned}$$

115 $$\begin{aligned} \Longrightarrow \Theta _{s} = (sin(s)+cos(y)+sin(z))e^{t}- \Theta _{y}, \end{aligned}$$

The PDE is solved with the aid of Maple software, leading to the following solution that contains an undetermined function.

116 $$\begin{aligned} \Theta (x,y,z,t,s) =(-cos(s)+sin(y)+ysin(z))e^{t}+{\mathcal {H}} (x, s-y, z, t), \end{aligned}$$

where, ${\mathcal {H}}(x, s-y, z, t)$ represents an undetermined function. By considering different scenarios, explicit forms of this function can be derived, with each case leading to a distinct particular solution.

Case 4.3.1:

Consider the invariant solution with the unidentified function ${\mathfrak {h}}(x)$ is given by,

117 $$\begin{aligned} \Theta (x,y,z,t,s) = (-cos(s)+sin(y)+ysin(z))e^{t}+ {\mathfrak {h}}(x). \end{aligned}$$

By substituting this invariant solution into Eq. (1) and the result is obtained in the form of ODE.

118 $$\begin{aligned} \beta e^{t}\left( \frac{d^{2}}{dx^{2}}{\mathfrak {h}}(x)\right) (cos(y)+sin(z)+ycos(z)+sin(s))+e^{t}(cos(y)+sin(z)+ycos(z)+sin(s))=0, \end{aligned}$$

The ODE is solved with the help of Maple software, and the solution for ${\mathfrak {h}}(x)$ is obtained as:

119 $$\begin{aligned} {\mathfrak {h}}(x)=-\frac{x^{2}}{2\beta }+{\mathscr {E}}_{1}x+{\mathscr {E}}_{2}. \end{aligned}$$

The specific solution is now obtained by combining Eq. (119) with Eq. (117).

120 $$\begin{aligned} \Theta (x,y,z,t,s) = (-cos(s)+sin(y)+ysin(z))e^{t}-\frac{x^{2}}{2\beta }+{\mathscr {E}}_{1}x+{\mathscr {E}}_{2}, \end{aligned}$$

that solution is exact solution.

**Fig. 12:**
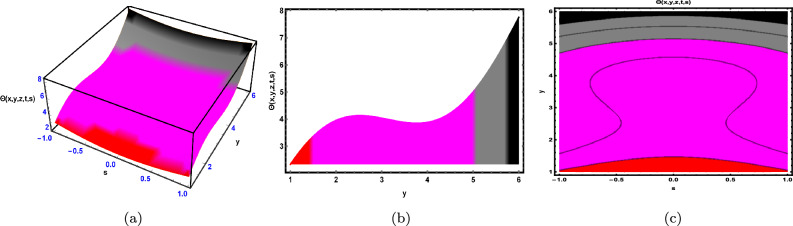
The solution for Eq. (120) of $$\Theta (x,y,z,t,s)$$ when $${\mathscr {E}}_{1} = -0.2,\; {\mathscr {E}}_{2} =0.5,\;$$$$t = 0.61,\; z = 0.95,\; x = -0.55,\; \beta = -0.77.$$

As shown in Fig. 12, the graphical representation of solution (120) for Case 4.3.1 is presented through 3D dynamics (Fig. 12a), 2D dynamics (Fig. 12b), and contour plots (Fig. 12c). The 3D dynamics (Fig. 12a) and contour plots [Fig. (12(c))] are generated over the intervals $-1 \le s \le 1$ and $1 \le y \le 6$, with the parameters ${\mathscr {E}}_{1},\; {\mathscr {E}}_{2},\; \beta$ and the variables $t,\; x,\; z$ fixed. The 2D dynamics (Fig. 12b) is obtained for $1 \le y \le 6$, where *s* is also fixed.

Case 5: The condition for the invariant surface in the fifth condition formulation is,

121 $$\begin{aligned} & \Gamma = \Upsilon ^{3}\frac{\partial \Theta }{\partial y}-\delta =0. \end{aligned}$$

Now, the following outcome may be achieved by altering the values of $\Upsilon ^{3}$ and $\delta$.

122 $$\begin{aligned} \Longrightarrow \Theta _{y}- {\mathcal {A}}(s, y, z, t) =0, \end{aligned}$$

it can now be written as,

123 $$\begin{aligned} \Theta _{y} = {\mathcal {A}}(s, y, z, t), \end{aligned}$$

where ${\mathcal {A}}(s, y, z, t)$ is a prescribed function. Eq. (123) denotes the governing PDE, and its solutions are obtained by considering different cases and assigning specific forms to the unknown functions.

Case 5.1:  The above equation becomes for ${\mathcal {A}}(s, y, z, t)\;=\;sin(s)+cos(y)+sin(z),$

124 $$\begin{aligned} \Theta _{y} = sin(s)+cos(y)+sin(z), \end{aligned}$$

The PDE is solved with the help of Maple software, producing the following solution that involves an arbitrary function.

125 $$\begin{aligned} \Theta (x,y,z,t,s) =sin(y)+(sin(s)+sin(z))y+{\mathcal {H}} (x, s, z, t), \end{aligned}$$

where ${\mathcal {H}}(x, s, z, t)$ denotes an undetermined function. By considering different scenarios, explicit forms of this function can be derived, with each case leading to a distinct particular solution.

Case 5.1.1: Consider the invariant solution involving the unknown function ${\mathfrak {h}}(x)$ is expressed as:

126 $$\begin{aligned} \Theta (x,y,z,t,s) = sin(y)+(sin(s)+sin(z))y+ {\mathfrak {h}}(x). \end{aligned}$$

By substituting this invariant solution into Eq. (1) and the result is obtained in the form of ODE.

127 $$\begin{aligned} \beta \left( \frac{d^{2}}{dx^{2}}{\mathfrak {h}}(x)\right) (sin(s)+cos(y)+sin(z)+cos(z)y+cos(s)y )=0, \end{aligned}$$

The ODE is solved with the assistance of Maple software, and the resulting form of ${\mathfrak {h}}(x)$ is given by:

128 $$\begin{aligned} {\mathfrak {h}}(x)={\mathscr {E}}_{1}x+{\mathscr {E}}_{2}. \end{aligned}$$

The specific solution is now obtained by combining Eq. (128) with Eq. (126).

129 $$\begin{aligned} \Theta (x,y,z,t,s) = sin(y)+(sin(s)+sin(z))y+{\mathscr {E}}_{1}x+{\mathscr {E}}_{2}, \end{aligned}$$

that solution is exact solution.

**Fig. 13:**
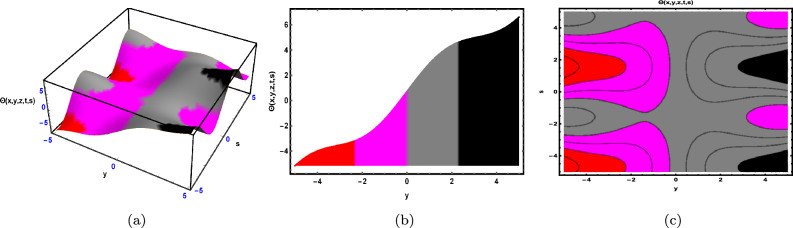
The solution for Eq. (129) of $$\Theta (x,y,z,t,s)$$ when $${\mathscr {E}}_{1} = -0.05,\; {\mathscr {E}}_{2} = 0.7,\;$$$$y = -0.5,\; z = .88,\; x = -0.5,\; \beta = 0.66.$$

As illustrated in Fig. 13, the graphical representation of solution (129) for Case 5.1.1 is presented through 3D dynamics (Fig. 13a), 2D dynamics (Fig. 13b), and contour plots (Fig. 13c). The 3D dynamics (Fig. 13a) and contour plots (Fig. 13c) are generated over the intervals $-5 \le s \le 5$ and $-5 \le y \le 5$, with the parameters ${\mathscr {E}}_{1},\; {\mathscr {E}}_{2},\; \beta$ and the variables $t,\; x,\; z$ fixed. The 2D dynamics (Fig. 13b) is obtained for $-5 \le y \le 5$, where *s* is also fixed.

Case 5.2:

The Eq. (123) becomes for ${\mathcal {A}}(s, y, z, t)\;=\;(sinh(s)+cosh(y)+sinh(z))e^{t},$

130 $$\begin{aligned} \Theta _{y} = (sinh(s)+cosh(y)+sinh(z))e^{t}, \end{aligned}$$

The PDE is solved with the aid of Maple software, resulting in the following solution that involves an undetermined function.

131 $$\begin{aligned} \Theta (x,y,z,t,s) =(sinh(y)+ysinh(s)+ysinh(z))e^{t}+{\mathcal {H}} (s, x, z, t), \end{aligned}$$

where ${\mathcal {H}}(s, x, z, t)$ represents an undetermined function. By considering different scenarios, explicit forms of this function can be determined, with each case leading to a distinct particular solution.

Case 5.2.1:  Consider the invariant solution with the unidentified function ${\mathfrak {h}}(x)$ is given by,

132 $$\begin{aligned} \Theta (x,y,z,t,s) = (sinh(y)+ysinh(s)+ysinh(z))e^{t}+ {\mathfrak {h}}(x). \end{aligned}$$

By substituting this invariant solution into Eq. (1) and the result is obtained in the form of ODE.

133 $$\begin{aligned} e^{t}\left( \beta \frac{d^{2}}{dx^{2}}{\mathfrak {h}}(x)+1\right) (sinh(s)+cos(y)+sinh(z)+ycosh(z)+ycosh(s))=0, \end{aligned}$$

The ODE is solved with the help of Maple software, and the solution for ${\mathfrak {h}}(x)$ is obtained as:

134 $$\begin{aligned} {\mathfrak {h}}(x)=-\frac{x^{2}}{2\beta }+{\mathscr {E}}_{1}x+{\mathscr {E}}_{2}. \end{aligned}$$

The specific solution is now obtained by combining Eq. (134) with Eq. (132).

135 $$\begin{aligned} \Theta (x,y,z,t,s) = (sinh(y)+ysinh(s)+ysinh(z))e^{t}-\frac{x^{2}}{2\beta }+{\mathscr {E}}_{1}x+{\mathscr {E}}_{2}, \end{aligned}$$

that solution is exact solution.

**Fig. 14:**
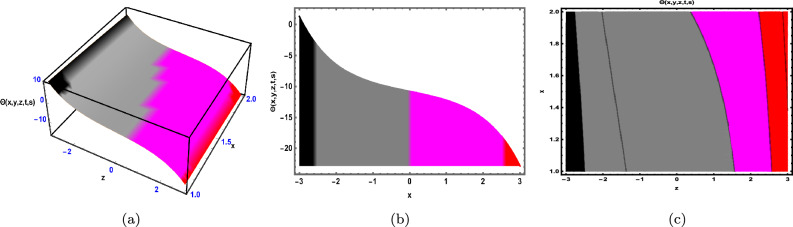
The solution for Eq. (135) of $$\Theta (x,y,z,t,s)$$ when $${\mathscr {E}}_{1} = -0.05,\; {\mathscr {E}}_{2} = 0.7,\;$$$$y = -0.5,\; t = .88,\; s = 0.66,\; \beta = 0.66.$$

As illustrated in Fig. 14, the graphical representation of solution (135) for Case 5.2.1 is presented through 3D dynamics (Fig. 14a), 2D dynamics (Fig. 14b), and contour plots (Fig. 14c). The 3D dynamics (Fig. 14b) and contour plots (Fig. 14c) are generated over the intervals $-3 \le z \le 3$ and $1 \le x \le 2$, with the parameters ${\mathscr {E}}_{1},\; {\mathscr {E}}_{2},\; \beta$ and the variables $t,\; y,\; s$ fixed. The 2D dynamics (Fig. 14b) is obtained for $1 \le x \le 3$, where *z* is also fixed.

Case 5.3:  The Eq. (123) becomes for ${\mathcal {A}}(s, y, z, t)\;=\;cosh(s)+sinh(y)+cosh(z),$

136 $$\begin{aligned} \Theta _{y} = cosh(s)+sinh(y)+cosh(z), \end{aligned}$$

The PDE is solved with the assistance of Maple software, leading to the following solution that contains an arbitrary function.

137 $$\begin{aligned} \Theta (x,y,z,t,s) =cosh(y)+(cosh(s)+cosh(z))y+{\mathcal {H}} (s,x, z, t), \end{aligned}$$

where ${\mathcal {H}}(s, x, z, t)$ denotes an unspecified function. By exploring different scenarios, particular forms of this function can be determined, with each case yielding a distinct solution.

Case 5.3.1:  Consider the invariant solution with the unidentified function ${\mathfrak {h}}(x)$ is given by,

138 $$\begin{aligned} \Theta (x,y,z,t,s) = cosh(y)+(cosh(s)+cosh(z))y+ {\mathfrak {h}}(x). \end{aligned}$$

By substituting this invariant solution into Eq. (1) and the result is obtained in the form of ODE.

139 $$\begin{aligned} \beta \left( \frac{d^{2}}{dx^{2}}{\mathfrak {h}}(x)\right) (cosh(s)+cosh(y)+cosh(z)+sinh(z)y+sinh(s)y )=0, \end{aligned}$$

The ODE is solved using Maple software, and the resulting solution for ${\mathfrak {h}}(x)$ is obtained as:

140 $$\begin{aligned} {\mathfrak {h}}(x)={\mathscr {E}}_{1}x+{\mathscr {E}}_{2}. \end{aligned}$$

The specific solution is now obtained by combining Eq. (140) with Eq. (138).

141 $$\begin{aligned} \Theta (x,y,z,t,s) = cosh(y)+(cosh(s)+cosh(z))y+{\mathscr {E}}_{1}x+{\mathscr {E}}_{2}, \end{aligned}$$

that solution is exact solution.

**Fig. 15:**
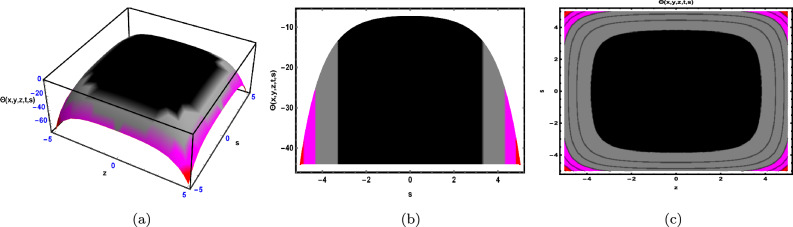
The solution for Eq. (141) of $\Theta (x,y,z,t,s)$ when ${\mathscr {E}}_{1} = -0.05,\; {\mathscr {E}}_{2} = 0.7,\;$ $y = -0.5,\; t = .88,\; x = 0.66,\; \beta = 0.66.$

As depicted in Fig. 15, the graphical representation of solution (141) for Case 5.3.1 is presented through 3D dynamics (Fig. 15a), 2D dynamics (Fig. 15b), and contour plots (Fig. 15c). The 3D dynamics (Fig. 15b) and contour plots (Fig. 15c) are generated over the intervals $-5 \le z \le 5$ and $-5 \le s \le 5$, with the parameters ${\mathscr {E}}_{1},\; {\mathscr {E}}_{2},\; \beta$ and the variables $t,\; y,\; x$ fixed. The 2D dynamics [Fig. (15(b))] is obtained for $-5 \le s \le 5$, where *z* is also fixed.

Remark:  All symbolic calculations were carried out in Maple, and all graphical simulations were produced in Mathematica.

## Conclusion

This study has presented a comprehensive nonclassical symmetry analysis of the (4+1)-dimensional Boiti–Leon–Manna–Pempinelli (4D-BLMP) equation, offering new insights into its nonlinear dynamics and associated wave structures. By formulating and solving systems of nonlinear determining equations, several novel nonclassical symmetries were identified. The classification of the resulting invariant functions enabled the construction of explicit exact solutions, thereby revealing a rich spectrum of wave phenomena. Graphical exploration of these solutions demonstrated the ability of the 4D-BLMP equation to generate diverse and non-singular structures, including complexiton-type solutions not typically found in related integrable models. These results highlight the distinctiveness of the 4D-BLMP framework and underscore the effectiveness of the nonclassical symmetry approach in uncovering new solution families. Overall, the findings advance the theoretical understanding of higher-dimensional integrable systems and provide potential tools for applications in physical sciences, where nonlinear wave dynamics play a central role. Future research may extend this analysis to perturbed or fractional versions of the BLMP equation, explore stability of the obtained solutions, or apply similar symmetry-based methods to other multi-dimensional nonlinear models.

## Data Availability

The datasets used and/or analysed during the current study available from the corresponding author on reasonable request.
